# Best practice indicators in workplace mental health promotion: development and expert validation

**DOI:** 10.1093/heapro/daaf147

**Published:** 2025-09-15

**Authors:** Carlos Montes, Artur Hernández, Carlos Dopico-Casal

**Affiliations:** Department of Social Psychology, Basic Psychology, and Methodology, University of Santiago de Compostela, Xosé María Suárez Núñez Street s/n, Campus Vida, 15782 Santiago de Compostela (A Coruña), Spain; Psychosocial Risks at Work Laboratory, Galician Institute for Occupational Safety and Health (Instituto de Seguridad y Salud Laboral de Galicia), Edificio SL 35, Avda. Fernando de Casas Novoa 35, 3rd Floor C-D, 15707 Santiago de Compostela (A Coruña), Spain; Department of Social Psychology, Basic Psychology, and Methodology, University of Santiago de Compostela, Xosé María Suárez Núñez Street s/n, Campus Vida, 15782 Santiago de Compostela (A Coruña), Spain; Psychosocial Risks at Work Laboratory, Galician Institute for Occupational Safety and Health (Instituto de Seguridad y Salud Laboral de Galicia), Edificio SL 35, Avda. Fernando de Casas Novoa 35, 3rd Floor C-D, 15707 Santiago de Compostela (A Coruña), Spain; Department of Social Psychology, Basic Psychology, and Methodology, University of Santiago de Compostela, Xosé María Suárez Núñez Street s/n, Campus Vida, 15782 Santiago de Compostela (A Coruña), Spain; Psychosocial Risks at Work Laboratory, Galician Institute for Occupational Safety and Health (Instituto de Seguridad y Salud Laboral de Galicia), Edificio SL 35, Avda. Fernando de Casas Novoa 35, 3rd Floor C-D, 15707 Santiago de Compostela (A Coruña), Spain

**Keywords:** mental health promotion, workplace, best practices, content validity, expert ratings

## Abstract

Most of our waking hours are spent in the workplace, where mental health significantly impacts workers’ quality of life and overall well-being. Mental health can have both positive and negative consequences not only on the working population but also on organizations and society as a whole. In this context, best practices in Workplace Mental Health Promotion (WMHP) are essential for guiding initiatives aimed at fostering mental health in occupational settings. The main objective of this study was to develop and validate best-practice indicators for WMHP. For this purpose, a qualitative empirical design was employed. Drawing on a review of the WMHP literature, a list of potential indicators was generated and subsequently submitted to an expert panel for evaluation. The reliability of the expert judgments was assessed using the intraclass correlation coefficient (ICC). Content validity was evaluated through the content validity ratio (CVR) and the modified kappa statistic (K*). As a result, 27 validated, evidence-based indicators were obtained, which showed consistency with findings from previous research. These results have important theoretical and practical implications and can inform future research as well as guide practitioners and organizations in the implementation of WMHP practices.

Contribution to Health Promotion

- The current study provides a comprehensive and validated set of indicators representing best practices in workplace mental health promotion.
- The evidence-based guidelines presented can support decision-making processes related to the design, implementation, and evaluation of mental health promotion interventions in occupational settings.
- The findings offer valuable guidance for organizations, occupational health and safety practitioners, and researchers in developing workplace initiatives aimed at preventing and managing work-related psychological health issues.

## INTRODUCTION

The workplace constitutes a major setting in which individuals spend a substantial portion of their lives. Recent international reports indicate that the average number of weekly hours worked per employed person worldwide was approximately 41.1 h (International Labour Organization 2024), with over one-third of all workers regularly working more than 48 h per week (International Labour Organization 2022). Since most of our waking hours are spent at work, this environment can significantly influence our overall health (for a review, see Niedhammer *et al*. 2021; Sonnentag *et al*. 2023), particularly our psychological well-being (for a review, see Harvey *et al*. 2017; Kelloway *et al*. 2023). Indeed, according to the European Agency for Safety and Health at Work (EU-OSHA; Leclerc *et al*. 2022), 27% of European workers report experiencing stress, depression, or anxiety that is caused or aggravated by their work.

Extensive research has found that adverse psychosocial working conditions can contribute to mental problems, including anxiety (Murcia *et al*. 2013), burnout (Shoman *et al*. 2021), and psychological distress (Nikunlaakso *et al*. 2023). A recent meta-review by Rugulies *et al*. (2023), synthesizing findings from seven systematic reviews and meta-analyses, revealed that individuals exposed to high job demands combined with low control, or high effort with low reward and low justice, have a 1.14–1.77 times greater likelihood of developing depression or taking sick leave due to psychological issues. However, employees’ mental health extends beyond individual well-being, exerting significant effects on organizations and society at large. For instance, poor mental health can reduce productivity, impair safety performance, increase sickness absence, and pose challenges in securing or maintaining employment (Farmer and Stevenson 2017; Rugulies *et al*. 2023; World Health Organization [WHO] 2022). Moreover, the societal costs associated with psychological problems at work are profound: globally, an estimated 12 billion workdays are lost annually due to depression and anxiety, corresponding to approximately $1 trillion in productivity losses per year (WHO 2024).

Given these substantial impacts, creating work environments that support mental health is essential for individual well-being, organizational effectiveness, and societal health. In this sense, Work Health Promotion (WHP) frameworks (e.g. Baranski 2001; Burton 2010; Leka *et al*. 2011; Motalebi *et al*. 2018) have emerged intending to improve “the health and well-being of people at work” (European Network for Workplace Health Promotion [ENWHP] 2018a, p. 2). Specifically, Work Mental Health Promotion (WMHP) initiatives have gained increasing attention as organizations recognize the need for targeted approaches to address employees’ psychological well-being (Czabała *et al*. 2011, Leka and Nicholson 2019, De Angelis *et al*. 2020, Arensman *et al*. 2022, Shiri *et al*. 2023, Waddell *et al*. 2023). For example, the Integrated Intervention Approach (LaMontagne *et al*. 2014) advocated for integrated interventions that, rather than focusing solely on risk reduction, also seek to enhance positive aspects of work and the individual worker capabilities. This approach has been widely adopted among different government agencies, including the Australian Government National Mental Health Commission (2023), the Centers for Disease Control and Prevention (2019), the National Institute for Health and Care Excellence (2022), and the World Health Organization (2022).

Overall, WMHP interventions are effective in improving psychosocial well-being. For instance, (i) comprehensive mental health programs (with evidence-based components, free or reduced-price access to therapy and medication, and referral to other programs if needed, among others) have been found to reduce symptoms of depression and anxiety by 35% and 37% respectively, while also improving productivity, reducing absenteeism by up to 0.7 working days per week, and increasing employee retention by 1.6 times (Ward *et al*. 2023); (ii) Employee Assistance Programs (EAPs), though often underutilized, have been associated with improvements in workers’ well-being (Wu *et al*. 2021); (iii) leadership training in mental health contributes to a more positive organizational climate and establishes communication channels to raise awareness of available mental health resources (Wu *et al*. 2021); (iv) leadership support interventions enhance the recognition of psychological issues and promote help-seeking behavior, thereby increasing the effectiveness of subsequent treatment (Wu *et al*. 2021); and (v) programs focused on monitoring and social support, combined with reduced working hours, facilitate return to work for employees recovering from mental health-related leave (Rugulies *et al*. 2023).

Despite these positive impacts, implementing WMHP interventions effectively remains challenging. The literature underscores the need for a more consistent evaluation of intervention outcomes to better understand what works and what does not (Goetzel and Ozminkowski 2008; Shaw *et al*. 2012). Key considerations include whether programs align with the organization's objectives, have management support, employ effective communication strategies, provide incentives for participation, effectively screen individuals at higher risk, and use evaluation indicators agreed upon by all stakeholders (Goetzel *et al*. 2014). On the other hand, different obstacles have been identified by both managers and employees. From the management's viewpoint, barriers include perceiving these programs as a luxury rather than an integral business component, concerns that they may distract workers from their daily responsibilities, or expectations of immediate return on investment (Goetzel and Ozminkowski 2008). From the employee perspective, difficulties include perceiving costs as prohibitive, difficulty integrating participation into the workday, and receiving information about available programs too late, among others (Noehammer *et al*. 2023). Additionally, stigma surrounding mental health issues may hinder employees’ willingness to communicate openly and seek help (Leclerc *et al*. 2022). Consequently, identifying best practices in WMHP is critical to ensure the effectiveness, sustainability, and integration of psychological interventions within organizational policies and practices, ultimately fostering healthier and more resilient work environments.

According to the ENWHP (2018b), a best practice in this context is defined as “a comprehensive Workplace Health Promotion strategy, providing suitable jobs for people with chronic illnesses and disabilities, in cooperation with health professionals, service providers, and other stakeholders” (para. 1). To recognize best practices in WHP, the ENWHP (n.d.) provides criteria grouped into six sectors for evaluating program quality based on the EFQM Business Excellence Model (Baranski 2001). Specifically, a best practice is characterized by (i) organizational management responsibility; (ii) promotion of worker participation in planning and implementation; (iii) grounding in a comprehensive understanding of health; (iv) reliance on accurate analysis and continuous improvement; (v) professional coordination with accessible information available to all employees at all times; and (vi) inclusion of specific indicators to measure impact.

Several international institutions have developed guidelines that provide fundamental frameworks for evidence-based recommendations and standards to promote psychological well-being at work. In addition to earlier reports from the EU-OSHA (Hassard *et al*. 2011) and the European Commission (Wynne *et al*. 2014), recent initiatives have made significant contributions to WMHP best practices. For example, the International Organization for Standardization (2021) developed ISO 45003, the first international standard focused on mental health, occupational safety, and well-being at work. This standard addresses key workplace mental health factors such as work-related stress, harassment, workplace violence, and other psychosocial issues. “WHO guidelines on mental health at work” (WHO 2022) outline a range of strategies applicable across multiple occupational levels, including organizational interventions, training for managers and workers, individual interventions, support for returning to work after mental health-related absence, and employment support for people living with psychological health conditions. Similarly, the “National Standard of Canada for Psychological Health and Safety in the Workplace” (Mental Health Commission of Canada 2022) is a voluntary standard that outlines requirements for a documented, systematic approach to developing and sustaining healthy and safe workplaces. It provides guidelines, tools, and resources to assist organizations in preventing mental harm and promoting psychological health at work.

In Spain, the National Institute for Safety and Health at Work (2022) created the “Healthy Company Awards” to recognize organizations making significant efforts to promote employee health and well-being. The evaluation criteria for this recognition include: (i) the company's conceptualization of WHP; (ii) leadership; (iii) participation and motivation; (iv) communication; (v) rationale; (vi) program multi-component nature; (vii) planning; and (viii) collaboration. Finally, the “Blueprint for Mentally Healthy Workplaces” (Australian Government National Mental Health Commission 2023) was developed to establish a nationally consistent approach for fostering psychologically healthy workplaces in Australia. It aims to align efforts among businesses, unions, government, workplace health, and mental health organizations around core pillars and principles supporting psychological well-being in occupational settings. These pillars include protecting workers from mental health risks, responding to signs of mental ill-health or distress, and promoting positive work aspects that contribute to optimal mental health.

Additional initiatives led by academics and researchers complement existing guidelines. For example, Wu *et al*. (2021) conducted a comprehensive review of literature published between 2000 and 2021 on mental health at work and EAPs within the North American context. Their review incorporated academic databases (e.g. Google Scholar, PsycINFO, SCOPUS) and corporate sources (e.g. “The Employee Assistance Trade Association [EASNA]; Health Enhancement Research Organization” [HERO]). The primary objective was to design the ”Mattingly Awards,” a joint initiative of the Johns Hopkins Bloomberg School of Public Health and The Luv u Project, aimed at promoting best practices in WMHP. Through this process, they identified eight criteria for best practices: (i) culture, (ii) robust mental health benefits, (iii) mental health resources, (iv) workplace policies and practices, (v) healthy work environment, (vi) leadership support, (vii) outcomes measurement, and (viii) innovation (for a detailed description of each category, see Wu *et al*. 2021).

### The present study

In recent years, significant efforts have been made in the field of WHP, including initiatives targeting mental health. Nevertheless, several limitations continue to affect the design, implementation, and generalizability of these interventions (e.g. Goetzel and Pronk 2010; Goetzel *et al*. 2014; Rugulies *et al*. 2023). Specifically, there remains a persistent need for more comprehensive knowledge regarding intervention effectiveness, identification of evidence gaps, and deeper evaluations of outcomes encompassing both financial and non-financial metrics (e.g. Goetzel and Ozminkowski 2008; Goetzel *et al*. 2014). Moreover, the applicability of WMHP programs is frequently constrained by several factors. First, organizations that do not clearly communicate their commitment to mental health risk fostering confusion and disengagement among employees; without visible and active organizational support, WMHP efforts are unlikely to succeed (Goetzel *et al*. 2014). Second, fragmentation of WMHP efforts can undermine their overall impact and relevance. When such programs are not fully integrated across all organizational levels—spanning human resources policies, organizational culture, and leadership practices—contradictions and perceptions of inadequate management commitment may arise, impeding acceptance and engagement (Shaw *et al*. 2012). Third, implementing rigorous methodological designs, such as randomized controlled trials, is often challenging in workplace settings due to ethical, logistical, and resource constraints (Rugulies *et al*. 2023). Fourth, cultural and structural barriers within organizations may impede the effective implementation of WMHP programs. These barriers include resistance to change stemming from established hierarchies, lack of support from middle management (Wu *et al*. 2021), and inadequate training of managers (Mental Health Commission of Canada 2022). Finally, limited financial and human resources may restrict organizations’ capacity to implement and sustain these programs (Goetzel *et al*. 2014; Rugulies *et al*. 2023).

Against this backdrop, the main goal of the present study is to review and validate a robust set of best practices criteria in WMHP to effectively manage and support employees’ mental health. This objective is particularly timely given the urgent need to address occupational risk interventions within a dynamic and volatile work environment (Frank *et al*. 2023), characterized by increasing telework, gender and racial disparities in employment, growing cultural diversity in teams, and the rise of precarious employment, among other challenges. To this end, a qualitative empirical study (Montero and León 2007) was conducted to identify and establish coherent standards for research and applied intervention in WMHP.

## MATERIALS AND METHODS

### Study population

The expert panel included members from the Work Group on Psychological Well-being of the Division of Work, Organizational, and Human Resource of the Spanish Psychological Association. To be considered eligible for inclusion, participants were required to have an established academic and/or professional trajectory in the field of Occupational Health Psychology and/or Occupational Risk Prevention at either the local or national level.

Initially, 15 experts consented to participate in the study. Of these, 5 were academic researchers (33.3%), and 10 were professional practitioners (66.7%) actively involved in consulting, training, and applied organizational psychology. The panel had an average of over 19 years of experience in the field, ranging from 4 to 35 years. Geographically, participants were drawn from various autonomous communities across Spain, including Madrid, Catalonia, Asturias, and Andalusia, among others, ensuring regional representation and contextual diversity.

One participant was excluded from the final analysis due to inconsistent response patterns. Specifically, this individual did not rate any indicator as essential, whereas the remaining panel classified on average 68.02% (SD = 14.45) of indicators as essential, with individual ratings ranging from 38.81% to 88.06%. Consequently, the final expert panel comprised 14 participants, whose collective expertise ensured a rigorous, practice-informed validation process grounded in both scientific and applied perspectives on workplace mental health.

### Literature review

An exhaustive review of relevant guidelines and scientific literature was conducted to develop a comprehensive set of criteria defining best practices for WMHP. This involved a systematic compilation and analysis of guidelines, standards, regulations, and academic and gray literature related to WMHP. Two major databases, PsycINFO and Web of Science, were searched using key terms including “best practices,” “mental health,” and “workplace.” To ensure the inclusion of comprehensive frameworks and recommendations, the search was restricted to peer-reviewed articles and grey literature (e.g. publications from governmental agencies or policy documents; Adams *et al*. 2017) published up to September 2023, in English or Spanish. Papers that dealt with overly specific aspects (e.g. what techniques work in particular contexts or enabling aspects for programs in particular populations) were excluded. Studies were excluded if they focused on highly specific elements (e.g. techniques effective only in certain contexts or for particular populations). In contrast, studies offering broad-based reviews of WMHP components or citing relevant guidelines or frameworks were retained. Additional sources were identified through manual searches and by reviewing the reference lists of eligible studies. These included documents from national and international organizations and governmental institutions.

The initial database search yielded three publications (Memish *et al*. 2017; Nexø *et al*. 2018; Wu *et al*. 2021). Titles and abstracts were then screened, followed by full-text assessments. Nexø *et al*. (2018) was excluded due to reliance on a single systematic review and concerns about evidence rigor. Memish *et al*. (2017) and Wu *et al*. (2021) were included; notably, Memish *et al.* provided a comparative evaluation of various initiatives and guidelines, selecting only those scoring above 50% on proposed indicators. Documents such as Leka *et al*. (2011) and Beyond Blue (2024) were excluded due to a narrow thematic focus.

Manual and reference list searches identified eight additional documents meeting the inclusion criteria. These comprised materials from national and international agencies (e.g. Knifton *et al*. 2011, ENWHP 2018c, National Institute for Safety and Health at Work 2022), international non-governmental organizations (WHO 2022), government-commissioned initiatives (Mental Health Commission of Canada 2022, Australian Government National Mental Health Commission 2023), and regulatory bodies in occupational health (Health and Safety Executive 2019; National Institute for Health and Care Excellence 2022). Thus, a total of ten publications were selected as foundational sources for developing best practice criteria in WMHP (Knifton *et al*. 2011, Memish *et al*. 2017, ENWHP 2018c, Health and Safety Executive 2019, Wu *et al*. 2021, Mental Health Commission of Canada 2022, National Institute for Health and Care Excellence 2022, National Institute for Safety and Health at Work 2022, WHO 2022, Australian Government National Mental Health Commission 2023).

### Development and refinement of the best practice indicators list

To establish a comprehensive framework for assessing best practices in WMHP, key thematic areas and relevant topics were first identified. These include core components such as risk identification and assessment, preventive measures, education and skills training, emergency management, program evaluation, and evidence-based planning. Based on the prior literature and guideline review, a list of 48 relevant criteria was formulated to encompass all identified areas. For each criterion, a precise definition was developed to clarify its meaning and operational scope. Subsequently, a set of specific indicators was identified for each criterion. These indicators were required to meet the following conditions: (i) objectivity and evidence-based foundation, (ii) grounding in reviewed documentation, (iii) alignment with WMHP goals, and (iv) capacity to facilitate compliance assessment.

To support expert evaluation, the initial set of 162 indicators underwent refinement by two independent reviewers. This involved the exclusion of redundant items, indicators with overlapping content, and those focused solely on aspects of legal compliance, which did not necessarily reflect best practice in mental health promotion. The refinement was conducted by two independent reviewers. Discrepancies were resolved via a third reviewer. This process yielded a streamlined list of 67 indicators, forming a robust and manageable set for expert validation.

### Procedure

Data for this study were collected through an online survey. Experts were contacted by email and provided with detailed information about the study's objectives, scope, and requirements. Prior to initiating the survey, informed consent was obtained from all participants. The consent process emphasized the voluntary nature of participation, as well as the confidentiality and anonymity of all responses. Participants were asked to rate each indicator, classifying it as (i) essential for defining best practices in WMHP, (ii) useful but not essential, or (iii) irrelevant. Additionally, participants were encouraged to provide qualitative feedback, including suggestions to enhance the relevance, clarity, or applicability of the indicators. The survey took approximately 20 minutes to complete. No incentives or compensation were offered for participation.

### Statistical analysis

The data analysis proceeded in three stages. First, inter-correlations among indicators were examined using Pearson's correlation coefficients, and intraclass correlation coefficients (ICC) assessed the reliability of expert judgments across the panel. Second, the content validity ratio (CVR) was calculated to measure the expert agreement on each indicator's essential status, using Lawshe’s (1975) formula:


(1) $$\mathrm{CVR}=\frac{\left(N_{e}-N/2\right)}{N/2}$$

where *N*_e_ is the number of experts rating the indicator as essential, and *N* is the total panel size. For a panel of 14 experts, a CVR >0.51 indicates an acceptable level of content validity at the 0.05 level of significance.

Third, the modified kappa statistic (K*), which adjusts for the probability of chance agreement, was computed. The formula used was:


(2) $$P_{c}=\frac{N!}{A!(N-A!)}\times 0.5^{N}\to K^{*}=\frac{\mathrm{RVC}-P_{c}}{1-P_{c}}$$

where *P*_C_ is the probability of chance agreement, *N* is the number of experts, and *A* is the number of experts who rated the indicator as essential. A K* value above 0.74 is considered to indicate excellent agreement among raters (Polit *et al*. 2007). All data analyses were performed using the IBM SPSS Statistics version 22.

## RESULTS

The reliability of expert judgements, based on the stability of the pooled assessments of all indicators, was 0.57 (95% CI for ICC = 0.41, 0.71), suggesting moderate consistency in the panel's responses (Anastasi 1988). The result was statistically significant (*P* < .001), indicating that this level of agreement was unlikely to have occurred by chance.

Next, the CVR was calculated for each of the 67 indicators. In a 14-member expert panel, a CVR value of ≥0.51 is considered statistically significant, meaning the indicator must be rated as “essential” by at least 11 panelists. Based on this criterion, 27 out of the 67 indicators (40.29%) were validated (see Table 1 for a summary of validated indicators). Importantly, each section of the original form contained at least one indicator with a statistically significant CVR.

**Table 1.** Validated indicators and their CVRs.

Indicator	CVR
1. The program/intervention is an ongoing initiative, with a sustained, long-term commitment, rather than a single, time-limited action.	0.86
2. The continuity of the program/intervention is evaluated and determined on the basis of the needs identified and the results obtained.	0.86
3. The program/intervention is designed within a comprehensive mental health concept (risk prevention, mental health promotion, and intervention in mental health problems).	0.71
8. Management is committed to making psychological health and safety part of the organization's decision-making process.	0.71
10. The program/intervention incorporates mental health as an integral part of the organization's human capital development strategies.	0.57
14. The program/intervention provides communication channels for workers or their representatives to express their concerns without fear of negative consequences, guaranteeing a safe environment in which to do so.	0.86
18. Program/intervention objectives are specific, measurable, realistic, and contextualized in time and place.	0.69
32. External collaborators, if present, have the knowledge, skills, and abilities required to perform their roles.	0.71
33. The program/intervention ensures that all workers have the necessary skills to perform their duties or are provided with opportunities to acquire them.	0.57
34. The program/intervention facilitates access to mental health resources.	0.57
37. The program/intervention includes specific actions for the return to work of workers coming back after a period of sick leave or absence due to mental health problems.	0.71
39. The program/intervention gathers information through qualitative or quantitative methods, or a combination of both.	0.57
41. The actions of the program/intervention do not focus exclusively on psychosocial risks and mental disorders but also consider positive/protective factors for mental health.	0.57
43. Program/intervention actions include measures that promote healthy behaviors.	0.71
44. The program/intervention includes measures that allow workers to mitigate stress related to their personal and family situations.	0.71
46. The program/intervention includes strategies to reduce the stigma toward mental health in the organization.	0.71
49. The program/intervention includes specific mental health training actions for management and middle management.	0.57
50. The program/intervention includes periodic mental health training actions for management and middle management.	0.57
51. The program/intervention includes awareness-raising actions to improve workers’ own awareness of well-being and recognize when and how to ask for help.	1
52. The program/intervention promotes awareness among workers about how psychological well-being at work can be influenced by external factors, such as physical health and personal relationships.	0.57
57. The program/intervention has a specific procedure for managing mental health-related incidents, such as traumatic events or fatalities (including suicide or attempted suicide, and disorders), where the roles and responsibilities of the parties involved in the investigation process are clearly established.	0.57
58. The program/intervention has a specific procedure for investigating mental health-related incidents, such as traumatic events or fatalities (including suicide or attempted suicide, and disorders), where the immediate and underlying causes of incidents are identified, corrective and preventive actions are implemented, and a measure of the effectiveness of all those implemented actions is made.	0.57
62. The evaluation of the results of the program/intervention includes the use of objective indicators to measure the effects of the actions carried out.	0.71
63. The evaluation of the results of the program/intervention incorporates indicators intended to measure the effects on the promotion of mental health in the short, medium, and long term.	0.57
64. The program/intervention evaluates the execution of the intervention, ensuring compliance with the initial guidelines and objectives, as well as the effectiveness and continuity of the implemented actions.	0.86
65. The program/intervention has procedures to record the actions carried out and the plans developed, thus ensuring the measurement of fidelity throughout the process.	0.71
67. The program/intervention makes mechanisms available to all stakeholders to ensure that they receive feedback on the results.	0.57

Only one indicator achieved unanimous agreement (CVR = 1.00): i51, which stated “The program/intervention includes awareness-raising actions to improve workers’ own awareness of well-being and recognize when and how to ask for help.” This highlights experts’ shared view on the crucial role of empowering employees to recognize mental health needs and seek support.

Among the remaining indicators, 4 demonstrated particularly high agreement (CVR = 0.80–0.99). Together, these indicators reflect a cohesive framework that emphasizes the need for strategic continuity (i1), evidence-informed planning (i2), psychological safety (i14), and systematic evaluation for fidelity and effectiveness (i64).

Nine additional indicators fell within the 0.70–0.79 range, reflecting robust—though slightly less unanimous—expert endorsement. While these indicators span different dimensions of intervention design, three overarching themes emerged from their content: (i) comprehensive conceptualization of mental health (i3, i43, i44, and i46), (ii) organizational and human capital readiness (i8, i32, and i37), and once again, (iii) robust monitoring and evaluation (i62 and i65).

Thirteen additional indicators met the minimum CVR threshold (≥0.51 and <0.70), reflecting statistically significant consensus among experts, albeit with greater variability in individual responses. Despite this variation, these indicators continued to map meaningfully onto the predefined thematic categories and served to reinforce the multidimensional nature of effective WMHP strategies. Several clustered within the theme of strategic integration and evidence-based planning, emphasizing the need for mental health initiatives to be embedded within human capital strategies (i10), structured through measurable objectives (i18), and informed by robust, mixed-methods data collection and evaluation practices (i39 and i63). Indicators i33, i49, and i50 highlight the importance of leadership training and individual skill development, pointing to the role of organizational leaders in cultivating psychologically safe environments through competence, awareness, and behavioral modeling. The indicators i34, i41, and i52 reflect a holistic understanding of well-being, encouraging organizations to move beyond narrow definitions of mental health to encompass emotional, social, and organizational determinants. Meanwhile, i57 and i58 center on emergency preparedness and crisis response, advocating for clear, proactive protocols to manage psychological emergencies in the workplace. Lastly, i67 emphasizes stakeholder communication and feedback mechanisms, ensuring that information dissemination is accompanied by structured opportunities for employee input, fostering transparency, accountability, and participatory implementation.

In contrast, 31 indicators fell into the 0.00–0.50 interval, indicating partial agreement among panelists. These include i4, i5, i6, i9, i11, i12, i13, i15, i16, i17, i20, i21, i22, i23, i26, i27, i28, i29, i31, i35, i38, i40, i47, i48, i53, i54, i55, i56, i59, i60, and i61. Finally, eight indicators showed negative CVR values, ranging from −0.14 to −0.57, showing that fewer than half of the experts rated them as essential (i7, i19, i24, i25, i30, i36, i42, and i45).

To further verify content validity, the modified kappa statistic (K*) was calculated for indicators with significant CVRs. K* values ranged from 0.55 to 0.99, with a mean of 0.66, indicating good agreement after adjusting for chance (Polit *et al*. 2007). Overall, these findings support the validity of the selected indicators as appropriate benchmarks for best practices in WMHP.

## DISCUSSION

The main objective of the study was to develop and validate robust indicators for workplace mental health promotion, grounded in scientific evidence and expert consensus from occupational health and business management professionals. To achieve this, a thorough review of the scientific literature and technical guidelines was conducted to construct a comprehensive set of best practice indicators. These indicators were then subjected to expert judgement. As a result, 27 indicators addressing specific field needs were identified as essential (Czabała *et al*. 2011, Shaw *et al*. 2012, Goetzel *et al*. 2014, LaMontagne *et al*. 2014, Wu *et al*. 2021, WHO 2022, Rugulies *et al*. 2023). More specifically, it was found that out of the 67 proposed indicators: (i) 88.05% were considered essential by at least half of the experts consulted; (ii) only 11 indicators were considered irrelevant by at least one panelist, and none were considered irrelevant by more than two panelists; and (iii) the evaluation of the results must take into account the method used to obtain them, since, when calculating the content validity using Lawshe’s (1975) method, indicators rated “useful but not essential” and “irrelevant” are given the same weight. This makes the procedure excessively strict and demanding (Choragwicka and Moscoso 2007), penalizing indicators that are not deemed “essential” to a large extent.

One of the main findings of this study is that experts reinforced the need for an integrative, holistic approach to WMHP. These interventions should be designed within a comprehensive framework to better prevent, protect, promote, and support the psychological well-being of employees (WHO 2022). In particular, rather than focusing exclusively on reducing occupational risks and managing mental problems, WMHP initiatives should also adopt a proactive perspective that fosters a supportive work environment and enhances workers’ resources (LaMontagne *et al*. 2014, Centers for Disease Control and Prevention 2019, Health and Safety Executive 2019, National Institute for Health and Care Excellence 2022, WHO 2022, Australian Government National Mental Health Commission 2023). Recent European initiatives, such as MENTUPP (Arensman *et al*. 2022) and H-WORK (De Angelis *et al*. 2020) projects, have employed this integrated approach to create mentally healthy workplaces. Findings from a MENTUPP pilot study revealed significant improvements in mental well-being and symptoms of anxiety at the 6-month follow-up (Tsantila *et al*. 2023).

Another crucial aspect identified by the expert panel was the need for policies and management commitment to integrate psychological health and safety into the organization's core functioning. Workplace mental health interventions are not isolated initiatives; rather, they should be seen as a central part of the organization's broader human capital development strategy and decision-making process (Wu *et al*. 2021, Mental Health Commission of Canada 2022, National Institute for Health and Care Excellence 2022). To ensure this, it is essential that organizations move beyond short-term efforts and commit to long-term, sustainable actions that incorporate mental health practices into daily operations and organizational routines (Mental Health Commission of Canada 2022; National Institute for Safety and Health at Work 2022; WHO 2022). Active support and sustained commitment to mental health by the organization can effectively foster an environment where mental health and well-being are prioritized (Goetzel *et al*. 2014), yielding lasting positive effects on workforce engagement. For instance, longitudinal evidence suggests that organization's commitment to mental health can positively influence team participation in decision-making and reflexivity, as workers dedicate time to rethink objectives, methods, and communication, underscoring the mediating role of leadership (Volpi *et al*. 2023).

The results of the current study also highlighted the importance of managers’ and leaders’ training and support in WMHP, aligning with previous research and best practice recommendations (e.g. Shaw *et al*. 2012, ENWHP 2018c, Wu *et al*. 2021, National Institute for Health and Care Excellence 2022, National Institute for Safety and Health at Work 2022, WHO 2022, Rugulies *et al*. 2023). Managers and leaders play an essential role in shaping organizational culture, fostering a health-promoting climate, and ensuring the effective implementation of workplace mental health policies and interventions (Kane-Urrabazo 2006; Milner *et al*. 2015; Wu *et al*. 2021). With adequate training in workplace mental health, managers and leaders can improve their knowledge, attitudes, and self-reported behavior in supporting employees experiencing psychological problems, recognize early warning signs of distress, and facilitate access to mental health resources (Gayed *et al*. 2018, Dimoff and Kelloway 2019, WHO 2022).

Furthermore, leaders who have previously demonstrated engagement in workers’ health promotion are more likely to gain employees’ trust, encourage their participation in health initiatives, and generate an open environment where workers feel comfortable discussing adverse working conditions and actions to improve their well-being (Nielsen *et al*. 2021). This is particularly relevant given that nearly four in 10 workers across the European Union report that they would not feel comfortable speaking to their manager or supervisor about their mental health (Leclerc *et al*. 2022). Research suggests that health-oriented leadership interventions can significantly improve exhaustion tendency, self-reported sickness absence, work-related sickness absence, and job satisfaction compared to nonintervention conditions (for a review, see Dannheim *et al*. 2022).

At the employee level, a number of indicators were considered essential by most experts. The highest agreement was achieved regarding the inclusion of awareness-raising actions aimed at improving workers’ own awareness of well-being and their ability to recognize when and how to ask for help. Training workers in mental health literacy and awareness aims to improve knowledge about mental health, reduce stigmatizing attitudes, and enable employees to support themselves or colleagues appropriately (Health and Safety Executive 2019; WHO 2022). Furthermore, this can have positive organizational effects as “increasing awareness of work-related influences on mental health, and the growing recognition of the need for ‘psychologically safe’ work may help to drive organizational efforts to improve psychosocial working conditions” (LaMontagne *et al*. 2014, p. 8).

Likewise, a substantial portion of expert judgements converged on other components that WMHP actions should incorporate, such as communication channels for workers to express their concerns without fear of negative consequences (National Institute for Safety and Health at Work 2022, Australian Government National Mental Health Commission 2023); adaptations in the return-to-work procedures after sick leave (Shaw *et al*. 2012; WHO 2022); strategies to mitigate stress related to personal and family circumstances (Health and Safety Executive 2019); strategies that promote healthy behaviors (Mental Health Commission of Canada 2022; National Institute for Health and Care Excellence 2022; National Institute for Safety and Health at Work 2022; WHO 2022); and strategies to reduce stigma toward mental health (Wu *et al*. 2021, WHO 2022, Australian Government National Mental Health Commission 2023).

In addition to the practices that shape the design, scope, and contents of WMHP initiatives across the different organizational levels, the expert panel emphasized the importance of properly evaluating program outcomes. Specifically, the use of objective impact measures is crucial to assess the real impact of implemented actions on employee and organizational well-being (ENWHP 2018c; Mental Health Commission of Canada 2022). Commonly used health measures in workplace mental health interventions include burnout, work engagement, perceived stress, psychological well-being, absenteeism rates, sickness absence, improvement in stressful conditions, level of relevant risk factors, and the number of suggestions for improvement submitted and implemented (ENWHP 2018c; Shiri *et al*. 2023; Waddell *et al*. 2023).

Moreover, the findings highlighted the need to assess not only intervention outcomes but also their implementation process, ensuring compliance with evidence-based guidelines and objectives (Mental Health Commission of Canada 2022). One suggested approach is to conduct internal audits (Mental Health Commission of Canada 2022), which play a fundamental role in occupational health and safety management (Jespersen *et al*. 2016). These audits might provide a quality management framework to obtain objective evidence about the intervention (e.g. effects, gaps between planned and actual implementation, corrective actions) and evaluate it to determine the extent to which audit criteria are fulfilled (International Organization for Standardization 2018; Lenning and Gremyr 2022). Such elements should ultimately guide decisions regarding the continuation of WMHP interventions, with particular attention given to both their outcomes and their effectiveness in addressing the identified needs (National Institute for Safety and Health at Work 2022).

Overall, the findings of this research address some of the main limitations that have affected the design, implementation, and generalization of WMHP interventions, as previously reported in the literature (Goetzel and Pronk 2010, Shaw *et al*. 2012, Goetzel *et al*. 2014, Wu *et al*. 2021, Mental Health Commission of Canada 2022, Rugulies *et al*. 2023). In particular, the present study provides a set of comprehensive and validated best practice indicators to develop WMHP initiatives that are not only theoretically sound but also offer actionable insights for organizations aiming to enhance workers’ well-being.

### Theoretical and practical implications

This work has important theoretical and practical implications. At the conceptual level, the indicators obtained can guide academics and/or researchers interested in exploring the factors that may differentially influence WMHP programs, as well as the conditions that act as obstacles or facilitators for their effective implementation. Furthermore, framing the findings within the Integrated Intervention Approach (LaMontagne *et al*. 2014) may facilitate the development of future research aligned with a holistic conception of mental health, since this model is frequently used in guidelines and recommendations from international bodies such as, e.g. the Australian Government National Mental Health Commission (2023) or the WHO (2022).

From an applied perspective, it provides practitioners with up-to-date, standardized, and evidence-based indicators to guide WMHP programs aimed at creating and maintaining a mentally healthy work environment (Rugulies *et al*. 2023). They also enable consistent comparisons across interventions and help identify key factors that affect program effectiveness (Shaw *et al*. 2012, LaMontagne *et al*. 2014, Frank *et al*. 2023). Finally, it addresses some critical needs in this field, such as reducing the overabundance of information available to organizations (Rugulies *et al*. 2023).

### Limitations and future research

Although this work contributes to increasing visibility of WMHP and the standardization of best practices in organizations, several limitations should be addressed in future research. The selection of the panelists was restricted to academics and professionals, and although they all had experience in the field of Occupational Health and/or Occupational Risk Prevention, direct experience working exclusively in WMHP was not required. As a follow-up to this study, it would be beneficial to hold discussions with experts specifically focused on WMHP to further adapt the proposed indicators to diverse organizational environments. Including professionals who work exclusively in WMHP in future expert panels would also strengthen the validation process. Moreover, future initiatives seeking recognition as best practices in WMHP could be assessed using the indicators developed here, providing additional validation for these findings.

Further, although the conceptual frameworks that informed the development of the indicators are designed for international applicability, the validation process was conducted with experts based in Spain. Nonetheless, cultural, legal, and organizational factors can influence how mental health promotion is perceived and operationalized (Pega *et al*. 2022, WHO 2022, Rugulies *et al*. 2023). For instance, workplace stigma, employment law, and occupational health infrastructure may vary substantially between countries or even industries. Therefore, while the indicators provide a useful starting point, we recommend that organizations contextualize them to align with local norms, labor policies, and cultural expectations. Future research could explore cross-national validation to assess the transferability and adaptability of the indicators in diverse global settings.

Besides, while the indicator development was informed by an extensive literature review, including both peer-reviewed and gray literature, we did not conduct a formal appraisal of the methodological quality or evidence level of the included sources. As such, the strength of the evidence underpinning individual indicators may vary. Future studies could enhance the rigor of indicator development by applying established quality assessment tools (e.g. GRADE, AMSTAR) to systematically evaluate the literature used in generating best practice indicators.

Finally, no guidance was developed on how organizations should implement the proposed indicators, representing a practical limitation. Thus, an important next step would be to translate the validated indicators into practical tools that facilitate their implementation in workplace settings. Developing a roadmap, checklist, or implementation guide could greatly enhance the usability and impact of the indicators. Such tools would help organizations, practitioners, and policymakers operationalize best practices by providing clear, actionable steps tailored to different organizational contexts (Jabali *et al*. 2024). Future research should therefore focus on co-designing and pilot-testing these implementation aids with key stakeholders to ensure they are both feasible and effective in real-world settings. This would bridge the gap between indicator validation and practical application, ultimately supporting broader adoption of evidence-based WMHP practices.

Related to this, future research should also aim to develop an official, standardized guide to best practices in WMHP, helping organizations navigate the overwhelming amount of information currently available (Rugulies *et al*. 2023). The challenges of conducting scientific trials in workplace settings (Rugulies *et al*. 2023) and the wide diversity of organizational environments—ranging from available resources to specific needs—highlight the necessity for robust methods to evaluate, compare, and identify effective interventions, which motivated the present study. The creation of new evaluation tools and methodologies has been previously identified as a key need in the field (LaMontagne *et al*. 2014, Frank *et al*. 2023, Rugulies *et al*. 2023). In addition, research that explores the financial impact of WMHP interventions recognized as best practice could encourage greater engagement from company management in supporting mental health in the workplace.

## CONCLUSION

The work environment is a key determinant of mental health, which has crucial impacts ranging from the individual to the organizational and societal levels. From an organizational perspective, mental health problems can result in decreased performance and irreversible productivity loss (WHO 2022, 2024). Conversely, developing health resources and promoting protective factors have been shown to benefit both workers (Wu *et al*. 2021, Rugulies *et al*. 2023, Ward *et al*. 2023) and organizations (Aldana 2001; Chapman 2012; Goetzel *et al*. 2014). Therefore, it is essential to establish evidence-based initiatives that promote mental health in the workplace to improve workers’ well-being and optimize overall organizational performance. In this context, the present study validated 27 best practice indicators addressing needs identified in the literature.

Firstly, this paper responds to the need to provide organizations with validated, evidence-based guidelines and recommendations to help manage the overload of information currently available regarding WMHP programs (Rugulies *et al*. 2023). These indicators, derived from scientific and technical literature and validated by experts, provide a solid foundation for practitioners and researchers to guide their interventions and investigations. This can facilitate informed decision-making about what to include in WMHP initiatives, as well as the elements necessary for effective implementation and evaluation.

Secondly, more consistent evaluation of the impact of interventions is needed to build knowledge about their effectiveness (Goetzel and Ozminkowski 2008). Supporting this, the present research can facilitate comparisons between programs and their outcomes. For example, it can serve as a checklist to verify whether programs meet the criteria identified as best practices and to explore differences between interventions across organizations, thereby providing valuable insights for improving future occupational health strategies and policies.

Finally, having a specific set of indicators to identify best practices in WMHP addresses a major limitation of existing programs: the wide variability that makes effectiveness comparisons difficult (Shaw *et al*. 2012). This work offers a common standard for organizations by providing consistent, validated guidelines. If correctly implemented, these indicators are expected to have a significant positive impact on organizations.

While WMHP remains an emerging field, current evidence supports the view that mental health at work is a fundamental component that can be effectively promoted, with positive consequences for workers, organizations, and society at large. Despite its limitations, this paper addresses critical needs in the field and aims to serve as a foundation for future research and to encourage organizations to adopt best practices in mental health promotion.

## Data Availability

Access to the data mentioned in this paper can be granted upon reasonable requests to the authors.
